# Bioinformatics approaches for viral metagenomics in plants using short RNAs: model case of study and application to a *Cicer arietinum* population

**DOI:** 10.3389/fmicb.2014.00790

**Published:** 2015-01-27

**Authors:** Walter Pirovano, Laura Miozzi, Marten Boetzer, Vitantonio Pantaleo

**Affiliations:** ^1^Genome Analysis and Technology Department, BaseClear B. V.Leiden, Netherlands; ^2^Institute for Sustainable Plant Protection of National Research CouncilTorino, Italy; ^3^Institute for Sustainable Plant Protection of National Research Council, Bari Research UnitBari, Italy

**Keywords:** bioinformatics, chickpea, ancient varieties, plant viruses, reference sequences, *de novo* assembly

## Abstract

Over the past years deep sequencing experiments have opened novel doors to reconstruct viral populations in a high-throughput and cost-effective manner. Currently a substantial number of studies have been performed which employ next generation sequencing techniques to either analyze known viruses by means of a reference-guided approach or to discover novel viruses using a *de novo*-based strategy. Taking advantage of the well-known *Cymbidium ringspot virus* we have carried out a comparison of different bioinformatics tools to reconstruct the viral genome based on 21–27 nt short (s)RNA sequencing with the aim to identify the most efficient pipeline. The same approach was applied to a population of plants constituting an ancient variety of *Cicer arietinum* with red seeds. Among the discovered viruses, we describe the presence of a *Tobamovirus* referring to the *Tomato mottle mosaic virus* (NC_022230), which was not yet observed on *C. arietinum* nor revealed in Europe and a viroid referring to *Hop stunt viroid* (NC_001351.1) never reported in chickpea. Notably, a reference sequence guided approach appeared the most efficient in such kind of investigation. Instead, the *de novo* assembly reached a non-appreciable coverage although the most prominent viral species could still be identified. Advantages and limitations of viral metagenomics analysis using sRNAs are discussed.

## INTRODUCTION

Over the past years deep sequencing experiments have opened novel doors to reconstruct viral populations in a high-throughput and cost-effective manner (Barba et al., 2014; Massart et al., 2014). Currently a substantial number of studies have been performed which employ next generation sequencing (NGS) techniques to either analyze known plant viruses by means of a reference-guided approach or to discover novel plant viruses using a *de novo*-based strategy (Kreuze et al., 2009, 2013; Navarro et al., 2009; Szittya et al., 2010; Wu et al., 2010; Giampetruzzi et al., 2012; Loconsole et al., 2012; De Souza et al., 2013; Candresse et al., 2014; Seguin et al., 2014; Marais et al., 2015). Despite of the significant advances made by sequencing technologies only a few methods have been proposed to specifically analyze viral metagenomes, especially if compared to the number of tools designed for, e.g., bacterial metagenome analysis (Schloss et al., 2009; Huson et al., 2011). At least in part this can be attributed to the fact that most viruses are still undiscovered; it has been suggested that at present less than 1% of the extent of viral diversity has been explored (Mokili et al., 2012). Moreover, viral communities tend to be phylogenetically distant from each other and therefore virus discovery and reconstruction heavily relies on *de novo* approaches. Another hurdle resides in the fact that viral populations are highly heterozygous which is mainly due to the low fidelity of the viral polymerase. This leads inevitably to a high mutation rate and consequently high variation even within the same populations that comprise a viral quasispecies (Domingo et al., 2012). Assembly tools of short sequence reads such as De Bruijn graph-based methods (Zerbino and Birney, 2008) are in principle designed for linear assembly of less diverse haploid and diploid genomes. As a result the assembly of viral (meta)genomes often leads to a substantial amount of contigs with generally a very short average length. Thus subsequent amplification of the resulting fragments using traditional methods (such as PCR and Sanger sequencing) is often essential to extend the draft assembly.

Also it should be mentioned that the chance of properly reconstructing one or more viral taxonomies heavily depends on the quantity of viral genomes present in the input sample. Given that viruses cannot easily be isolated, generally a high sequencing coverage is necessary to pick up all relevant viral genomic material within a plant sample. Alternatively, virus enrichment is needed (Roossinck, 2012). In other words, projects that aim to characterize viral (meta)genomes in plants can become very costly as these are mostly based on sequencing total DNA or RNA libraries that contain only a small fraction of viral material.

The silencing-based antiviral plant response may help somehow in this deal; it implies the recognition of double-stranded (ds) or ds-like RNAs of viral origin by members of plant Dicers (DCLs; Aliyari and Ding, 2009). The recognized viral RNAs are then processed by DCLs into viral small interfering RNAs (v-siRNAs; reviewed by Ding and Voinnet, 2007 and Ruiz-Ferrer and Voinnet, 2009). Two distinct classes of v-siRNAs have been identified: primary v-siRNAs, which result from the DCL mediated cleavage of an initial trigger RNA, and secondary v-siRNAs, which require a plant RNA-directed RNA polymerase (RDR) for their biogenesis (Wassenegger and Krczal, 2006; Donaire et al., 2008; Ruiz-Ferrer and Voinnet, 2009; Vaistij and Jones, 2009; Garcia-Ruiz et al., 2010; Wang et al., 2010). The amplification and high level of v-siRNAs accumulation in many but not all virus infections depends on the combined activity of the host-encoded RDRs such as RDR1, RDR2, and RDR6 with other factors such as the RNA helicase SDE3. The amplification mechanism may result in production of secondary amplified v-siRNAs also in case of weakly induced silencing (i.e., low accumulation of viral RNAs; Garcia et al., 2012).

v-siRNAs can also be successfully used to cover known viral genomes by aligning reads to the reference sequences (ref_seq), thus providing a simple method for detection of known viruses and viroids and their variants (Navarro et al., 2009; Pantaleo et al., 2010). In addition, Kreuze et al. (2009) have used at first sRNA libraries for *de novo* reconstruction of the complete genome of a known plant RNA virus from multiple contigs of v-siRNAs. Moreover v-siRNAs can be used for non-homologous discovery of novel plant infectious entities (Wu et al., 2012). The deepness and the low level of bias of sRNAs are key factors for the success of either reference alignment and *de novo* assembly based approaches. Seguin et al. (2014) have demonstrated that is possible to reconstruct the entire genomic master sequence of DNA and RNA viruses from both model and crop plants using v-siRNA libraries when sequencing approximately 20 million deep sRNA libraries. Other research groups have spent efforts to demonstrate that bias in cloning procedures may hide some of the sRNAs and therefore they have studied and developed alternative strategies to reduce such bias (Sorefan et al., 2012).

In the present paper we analyze a specific sRNA library from leaves sampled within plants constituting a *Cicer arietinum* ancient variety (Red of Ruvo, Apulia-Italy) and from leaves of *Nicotiana benthamiana* plants infected with the *Cymbidium ringspot virus* (*CymRSV*) in a ratio of approximately 1000 to 1. The presence of v-siRNAs from *CymRSV* allows us to compare different bioinformatics tools developed for reference-guided or *de novo* assembly based approaches. Protocols were also applied to the viral metagenome of *C. arietinum*. We find that a reference-guided approach is very successful in the reconstruction of the most abundant viruses. Instead *de novo* approaches clearly suffer from the heterogeneity within viral populations. Among the discovered viruses, we describe the presence of a *Tobamovirus* referring to the *Tomato mottle mosaic virus (ToMMV;* NC_022230), which was not yet observed on *C. arietinum* and also not yet revealed in Europe, and one viroid referring to *Hop stunt viroid (HSVd;* NC_001351.1) never reported in chickpea. Accordingly, we discuss our findings and provide suggestions that aim to discover plant–viruses using a cost-effective approach based on sRNA sequencing.

## MATERIALS AND METHODS

### PLANT MATERIALS, VIRUS, RNA EXTRACTION, AND SMALL RNA SEQUENCING

The use of wild type* N. benthamiana* plants and infection with *CymRSV in vitro* transcripts was previously described (Pantaleo et al., 2007; Pantaleo and Burgyan, 2008). The plant growth chamber was set with 10 h in light and 14 h in dark at 22°C. Seed population constituting an ancient variety of *C. arietinum* named “Red Chickpea of Cassano delle Murgie” accession “Red of Ruvo” (in collection at Mediterranean Germplasm Database, http://ibbr.cnr.it/ibbr/resources/mediterranean-germplasm-database) was grown in an open air collection field. Plant leaf material representing the entire population (i.e., one leaf per plant covering the 30% of the plants) was collected at flowering stage and bulked. Total RNA was extracted from plant tissues using Tri-Reagent (SIGMA) following manual instructions. Low molecular weight RNA was enriched as previously described (Johansen and Carrington, 2001) and mixed in a ratio of 1000 (chickpea) to 1 (*N. benthamiana*) in amount. Subsequently, libraries of sRNAs were produced using a TruSeq Small RNA Sample Kit (Illumina) and sequenced with standard sequencing oligos on the Illumina HiSeq 2500 platform. Short sequence reads were generated using bcl2fastq software (v 1.8.3). The dataset has been deposited in GEO Omnibus under the entry code GSE63378.

### BIOINFORMATICS

Small RNA adapters were removed from the Illumina sequence reads using the “Trim sequences” option of the CLC Genomics Workbench (v 6.0.4). For the ref-seq based approach, the resulting sub-reads were aligned against the *CymRSV* (NCBI accession code NC_003532) and *ToMMV* (NCBI accession code NC_022230) reference genomes using the “Map reads to reference” option of the CLC Genomics Workbench (v 6.0.4). For the *de novo* based approach assemblies were generated, respectively, with Velvet version 1.2.10 (Zerbino and Birney, 2008), Oases version 0.2.08 (Schulz et al., 2012), and MetaVelvet (Namiki et al., 2012). Alignment of the assembled contigs against the *CymRSV* and *ToMMV* reference genomes was performed using Burrows-Wheeler Aligner (BWA) v. 0.7.7 (Li and Durbin, 2009). From the alignment consensus sequences were generated using SAMtools version 0.1.19 (Li et al., 2009). SNP detection was performed with Nucmer which is part of the MUMmer analysis package (version 3.22; Kurtz et al., 2004). Graphical alignment visualization were generated using the Integrative Genomic Viewer (IGV; Robinson et al., 2011). All software was used with default settings unless otherwise specified in text and figures.

## RESULTS

### SHORT RNA DATASET

**FIGURE 1:**
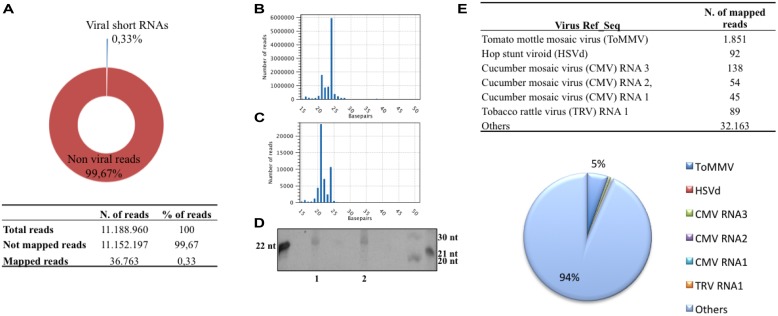
**Statistics of the short RNA library from *Cicer arietinum* ancient variety “Red of Ruvo.”** Pie graph and table resuming the dimension of the library and the fraction of sRNA reads mapping with plant virus ref_seq dataset **(A)**, distribution of sRNA length of non-viral reads **(B)** and viral reads **(C),** Ethidium bromide staining of sRNA from *C. arietinum* (1), not infected *Nicotiana benthamiana* (2; **D**), table and pie graph resuming viral reference sequence (ref_seq) represented in the sRNA library **(E)**.

sRNAs specifically of 20–27 nucleotides with 5′-phosphate and 3′-OH (likely to be DCL products) were isolated from *C. arietinum* “Red of Ruvo” and from a *CymRSV-*infected *N. benthamiana* and further identified by high-throughput Illumina sequencing. The library yielded in total more than 11 million reads (table in **Figure 1A**) with a minimum and maximum length of 16 and 27 nt (**Figure 1B**). A consistent fraction of these (approximately 6 million) were 24 nt in length (**Figure 1B**) and this is in line with observations in ethidium bromide staining of the polyacrylamide isolation gel (**Figure 1D**, lane 1). The abundance of 24 nt sRNAs found in chickpea also agrees with previous studies showing that in plants, except for a few species, the 24 nt sRNAs are more abundant than the 21 nt class (Rajagopalan et al., 2006; Moxon et al., 2008; Pantaleo et al., 2010). Accordingly, **Figure 1D** shows that the sRNAs from *C. arietinum* (lane 1) and not infected *N. benthamiana* (lane 2) equally migrate as they are of the same size.

Those sRNAs flanked by the 3′ and 5′ TrueSeq Illumina adapters were compared with a plant virus reference dataset (ftp://ftp.ncbi.nih.gov/refseq/release/viral/), which is defined by collection of 1.677 unique plant virus master sequences. In total 36.763 reads could be mapped to the plant virus reference dataset (excluding those from *CymRSV*), thus the v-siRNAs constituted only 0,33% of the entire library (**Figure 1A**). More than half of the v-siRNAs were of length 21 nt (ca. 23.000), whereas those of length 22 and 24 nt were less abundant (of ca. 7.000 and 11.000, respectively; **Figure 1C**). This distribution recapitulates what was previously observed in plant virus infections, particularly in those infected with RNA viruses, and it mirrors the plant DCLs activity involved in RNA-silencing-based antiviral activity (reviewed by Shimura and Pantaleo, 2011). The most represented viral genomes by viral reads comprise the *Tobamovirus ToMMV* (i.e., 1.851 reads), the *Hop stunt viroid (HSVd*; i.e., 92 reads), the *Cucumber mosaic virus (CMV*; i.e., 45, 54, and 138 reads map against CMV RNA1, 2 and 3, respectively) and the RNA 1 of the *Tobacco rattle virus (TRV*; i.e., 98 reads; Table in **Figure 1E**). The above mentioned viral reads all together represented about 4.5% of the entire population of siRNAs that map against the plant virus dataset, indeed most viral reads are scattered in exiguous number across viral ref_seq (i.e., less than 10 unique reads per ref_seq). Moreover, these putative viral siRNAs align to unrelated viruses (i.e., belonging to different viral families) thus not suggesting the need for further investigations.

### REFERENCE SEQUENCE-GUIDED ASSEMBLY

As mentioned above and detailed in the “Materials and Methods” section, the sRNA library under analysis included a small fraction of siRNAs from *CymRSV*-infected *N. benthamiana*. Thus, we have first reconstructed the *CymRSV* genome through alignment of the sRNA reads against its ref_seq (NCBI accession code NC_003532). The alignment statistics are shown in **Table 1**. A total of 364.590 sRNAs reads, with an average length of 21 nt, mapped onto the 4.733 nt long ref_seq. Each nucleotide of *CymRSV* was covered by sRNA reads 77,03 times on average and all together the reads were able to reconstruct 99% of the entire genome (the final consensus sequence comprises 4.698 of the original 4.733 nt). Subsequent variant calling revealed the presence of 13 SNPs; such degree of variability between the consensus sequence and the ref_seq is in agreement with previous findings for *Tombusvirus* variability at 3 days after inoculation of an *in vitro* transcript (Russo et al., 1994).

**Table 1:** sRNA alignment statistics against the *Cymbidium ringspot virus (CymRSV)* reference sequence (ref_seq) NC_003532.

Length of the reference CymRSV sequence NC_003532	4.733
Number of mapped reads	364.590
Average length	21,08
Average coverage	77,03
Number of nucleotides in consensus sequence	4.698
Fraction of reference covered	0,99
Number of SNPs with NC_003532	13

The same approach was used for the reconstruction of *ToMMV* (NCBI accession code NC_022230). This virus was the best represented by viral reads population in chickpea (**Figure 1E**). Individual alignment of all reads against exclusively NC_022230 shows that a total of 1.909 sRNAs (with an average length of 21,55 nt) could be mapped (**Table 2**). Given the reference length of 6.398 nt, each *ToMMV* nucleotide was represented 6,4 times on average; in total 87% of the entire genome was covered at least one time (the consensus sequence covered 5.582 out of 6.398 nt). Finally, the variant calling analysis revealed the presence of 39 SNPs. Notably, the number of SNPs found is sensibly higher than those found in the model system *CymRSV*. This is particularly interesting if we consider the incidence of SNPs in relation to the total *ToMMV* v-siRNAs (i.e., 1.909) versus those of *CymRSV* (364.590). Nonetheless, the large variability encountered in the present metagenomics investigations on field-cultivated plants is fully in line with previous reports for other non-*in vitro* plant/virus systems (Seguin et al., 2014).

**Table 2:** sRNA alignment statistics against the *Tomato mottle mosaic virus (ToMMV)* ref_seq NC_022230.

Length of the reference ToMMV sequence NC_022230	6.398
Number of mapped reads	1.909
Average length	21,55
Average coverage	6,4
Number of nucleotides in consensus sequence	5.582
Fraction of reference covered	0,87
Number of SNPs with NC_022230	39

**FIGURE 2:**
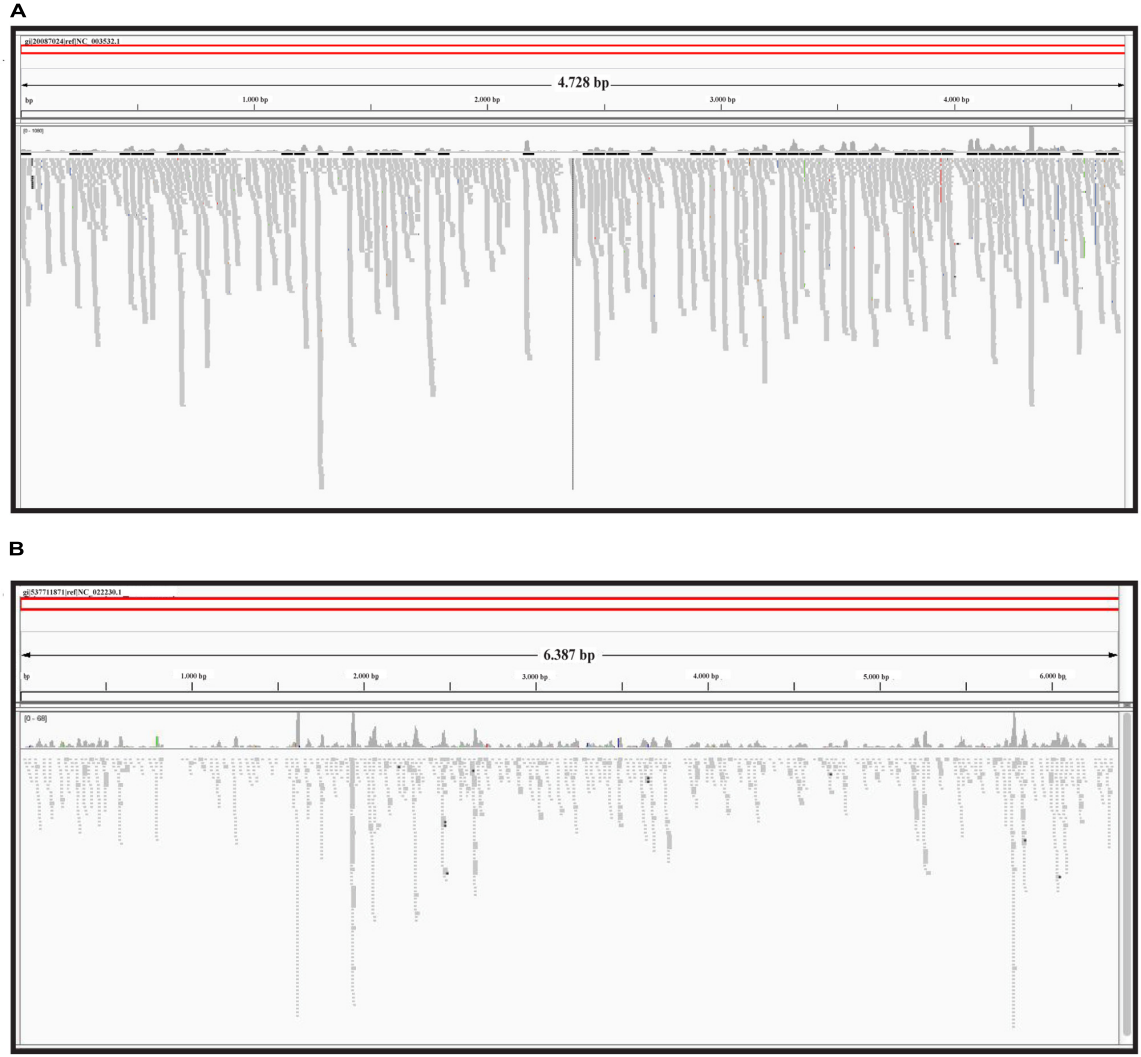
**Reference sequence based approach.** sRNAs alignment to the *CymRSV* ref_seq NCBI# NC_003532 **(A)** and to *ToMMV* ref_seq NCBI# NC_022230 **(B)**.

**FIGURE 3:**
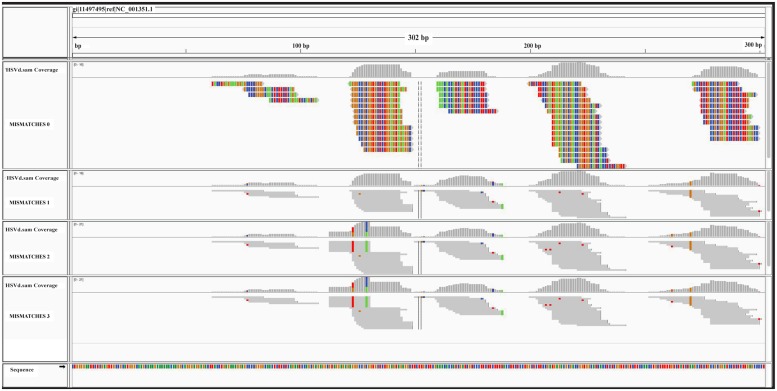
**Graphical overview of the ref_seq-based approach.** sRNA alignments against the HSVd ref_seq NCBI# NC_001351 are displayed. The four consecutive panels correspond to 0, 1, 2, and 3 mismatches used in BWA alignment. All nucleotides are colored in the expanded graphic representation (mismatches = 0): C in blue, G in brown, A in a green, and T in red. Mismatches are colored in not expanded representations.

The graphic distribution of mapped reads against *CymRSV* and *ToMMV* is reported in (**Figures 2A,B** respectively). The graphic representation shows that *ToMMV* is almost entirely covered by v-siRNAs in a manner that is at least visually similar to that of *CymRSV*, thus reproducing a high genome coverage of 99% (*CymRSV*) and 87% (*ToMMV*) already indicated in **Tables 1** and **2**. Also a viroid referring to *HSVd* (NC_001351.1) was almost entirely reconstructed with only 92 reads when applying the same settings as for *CymRSV* and *ToMMV* (**Figure 3**). The shortness (302 bases) of the viroid allowed us to check whether a better coverage could be obtained by introducing mismatches in BWA alignment protocol. Indeed, some gaps were covered when using two mismatches (**Figure 3**) and at three mismatches no further improvement was obtained. Still, the 5′ part of the viroid (position 1–60 of the released HSVd ref_seq NC_001351), upstream the central conserved domain (CCD; Keese and Symons, 1985; Visvader and Symons, 1985) could not be covered by introducing single variants in the alignment with the ref_seq (see Discussion).

### *DE NOVO* BASED APPROACHES AND v-siRNAs ASSEMBLY

Given that at present ca. 900 species of plant viruses have been determined (Ninth Report of the International Committee on Taxonomy of Viruses. Elsevier Academic Press, 2012) in most cases no good reference (or master consensus genome) is available. In this scenario *de novo* assembly of viral genomes should be considered as a valid alternative, thus allowing the creation of a consensus sequence set that best represents the underlying viral population with a non-homology approach. These consensus sequences can serve as a proper basis for reference alignment and variant calling as described above. At present De Bruijn graph-based algorithms (reviewed by MacLean et al., 2009) are the methods of choice to assemble a set of NGS reads. In brief the algorithm divides the NGS reads into short sub-reads (so-called *k*-mers) and subsequently it searches the ideal assembly path in the graph through overlap between the *k*-mers. Thus, the algorithm is optimized for a fast merging of millions of short NGS reads into (large) genomic fragments. In fact, De Bruijn graph based methods such as Velvet (Zerbino and Birney, 2008) and SOAP *de novo* (Li et al., 2010) are widely employed for the genomic assembly of prokaryotes and eukaryotes. The algorithm, however, appears to be less suited for the assembly of fragments with unbalanced coverage distributions such as generated in RNA-Seq and metagenomic libraries. In the latter case chromosomes of different microbes are present in a metagenomics sample proportional to their relative abundance. As such the relative frequencies of short reads covering the various nodes in the De Bruijn graph differ with respect to a standard linear genome assembly. To overcome these problems specific tools are developed for the assembly of transcriptomes, e.g., Trinity (Grabherr et al., 2011) and Oases (Schulz et al., 2012) and metagenomes [e.g., and MetaVelvet (Namiki et al., 2012) and Ray Meta (Boisvert et al., 2012)]. Potentially these methods could also be of good use for assemblies of viral metagenomes. We here apply the three well-used bioinformatics assembly tools (Velvet, Metavelvet, and Oases) and compare their relative ability to reconstruct the viral metagenome. Also we developed an in-house modification of the standard Velvet protocol where, prior to the assembly, duplicate reads are removed (REDREM) thus taking into account issues related to unbalanced genome coverage (see Discussion). All strategies were evaluated at different *k*-mer settings.

**FIGURE 4:**
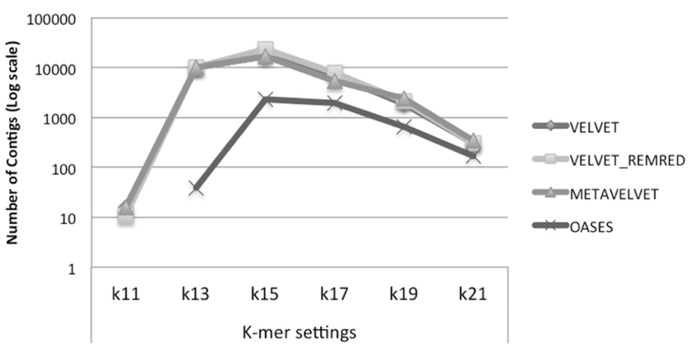
**Number of contigs (log scale) obtained using Velvet, Metavelvet, and Oases short (s)RNA assembly tools with different *k-*mer settings.** REMRED = Remove redundant.

**FIGURE 5:**
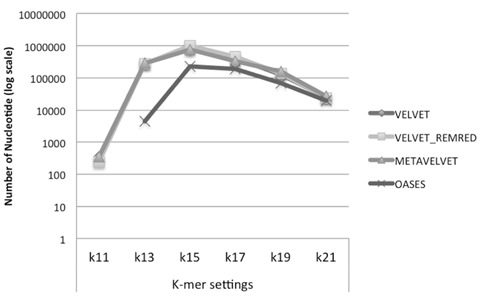
**Number of bases (log scale) in the assembled contigs obtained using Velvet, Metavelvet, and Oases short RNA assembly tools with different *k-*mer settings.** REMRED = Remove redundant.

We observe that Velvet and Metavelvet constructed the largest number of consensus sequences at all *k*-mer setting (hereafter “*k*”) used. Surprisingly, for all tools the maximum number of consensus sequences was obtained at setting *k* = 15 (**Figure 4**; Supplementary Data 1A). More specifically, Velvet is able to provide a higher number of consensus sequences at *k* = 15 when using a non-redundant sRNA dataset, i.e., 16.604 sequences using Velvet versus 23.251 using Velvet REDREM (Supplementary Data 1A,B, respectively). Accordingly, the total number of assembled nucleotides was higher when using Velvet and Metavelvet compared to Oases (**Figure 5**; Supplementary Data 1).

**FIGURE 6:**
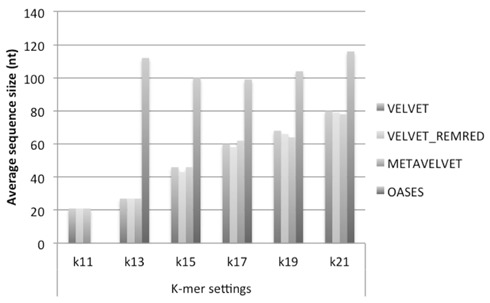
**Average size of contigs obtained using Velvet, Metavelvet, and Oases short (s)RNA assembly tools with different *k-mer* settings.** REMRED = Remove redundant.

On the other hand, when comparing the average size of the contig sequences obtained by the different methods at different *k*-mers’s, Oases appears to be the best method. Indeed, except in the case of *k* = 11, Oases appears to provide the longest consensus sequences on average for all settings (**Figure 6**). Regarding the consensus sequences generated with Velvet and Metavelvet, the average length increased from *k* = 11 (the lowest) to *k* = 21 (the highest; **Figure 6**). The longest consensus sequence was obtained by Oases at *k* = 17 (i.e., 919 nt in length, Supplementary Data 1). Other tools obtained their maximum length (between 400 to 600 nt) at *k*-values ranging from 15 to 21 (Supplementary Data 1).

### *DE NOVO* ASSEMBLY OF CymRSV AND ToMMV

**FIGURE 7:**
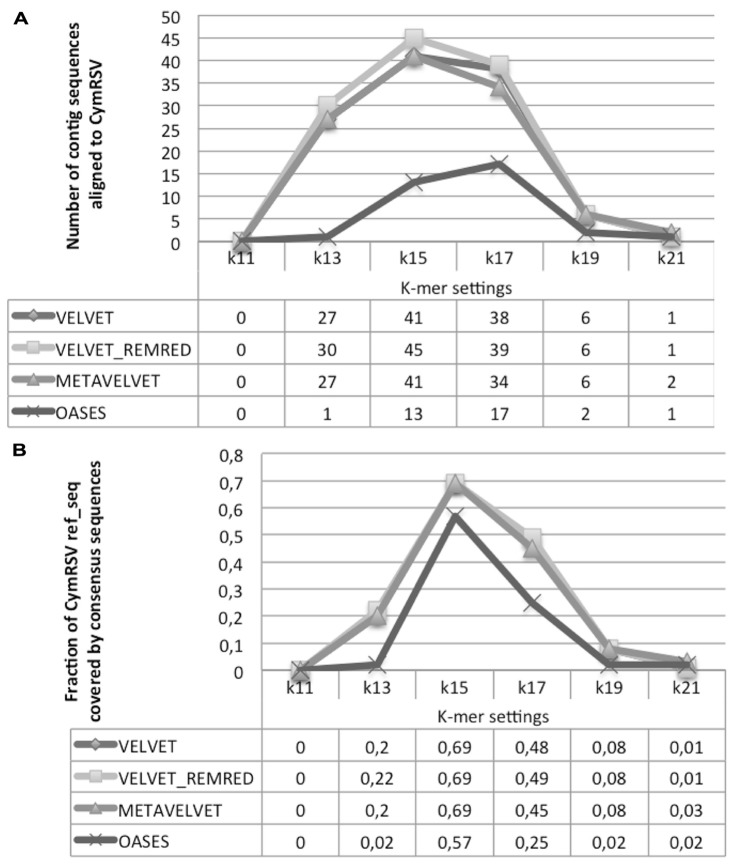
**Alignment statistics of contig sequences constructed using Velvet, Metavelvet, and Oases short RNA assembly tools with different *k-*mer settings.** The graph and table display the number of contig sequence aligning with *CymRSV* ref_seq **(A)** and fraction of CymRSV ref_seq covered by the consensus sequence **(B)**. REMRED = Remove redundant.

All contig sequences obtained by different tools and settings were aligned against the *CymRSV* ref_seq. First Velvet REMRED and second Metavelvet and Velvet showed to be the most efficient tools by generating, respectively, 45 and 41 consensus sequences at *k* = 15 (**Figure 7A**). For these approaches an increase or a decrease of *k* values resulted in a sensible decrease of contig sequences aligning with the *CymRSV* ref_seq: e.g., in the case of Velvet REMRED the use *k* = 13 or *k* = 17 reduces the number of contigs to 30 and 39, respectively, whereas for *k* = 15 in total 45 contigs were assembled (**Figure 7A**). Moreover, when applying *k* = 15 to both Velvet methods and MetaVelvet the coverage of the *CymRSV* genome was the highest, i.e., 0,69% (3.247 nt out of 4.733 nt of the *CymRSV* genome). Again a setting of *k* = 13 or *k* = 17 sensibly reduces the efficiency of the method (**Figure 7B**).

Oases reached a slightly lower coverage level (57%) at *k* = 15 (**Figure 7B**) although the average length of the 13 consensus sequences was significantly higher than the other three methods (**Figure 7A**).

Surprisingly, all assembly tools evaluated at *k* = 15 detected a similar number of SNPs (14 in case of the Velvet methods, 12 in case of Oases; see Supplementary Data 2), which is comparable to the number of SNPs detected with the reference-guided assembly (i.e., 13, **Table 1**). As previously underlined, the method of *CymRSV* inoculation and the timing of sampling may impede a further increase of variability within the viral genome (Russo et al., 1994).

**FIGURE 8:**
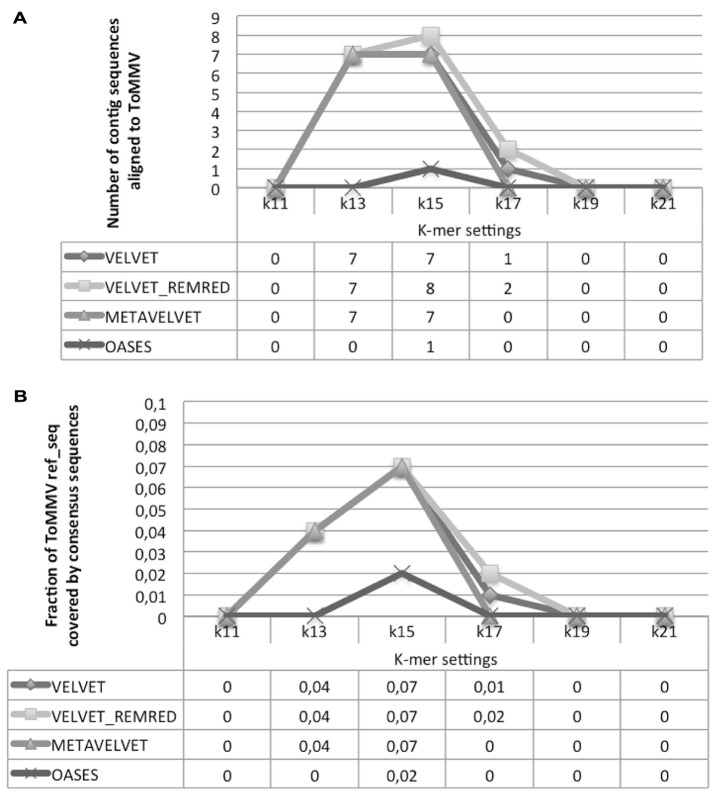
**Alignment statistics of contig sequences constructed using Velvet, Metavelvet, and Oases short RNA assembly tools with different *k-*mer settings.** The graph and table display the number of contig sequences aligning with ToMMV ref_seq **(A)** and the fraction of ToMMV ref_seq covered by the consensus sequence **(B)**. REMRED = Remove redundant.

**FIGURE 9:**
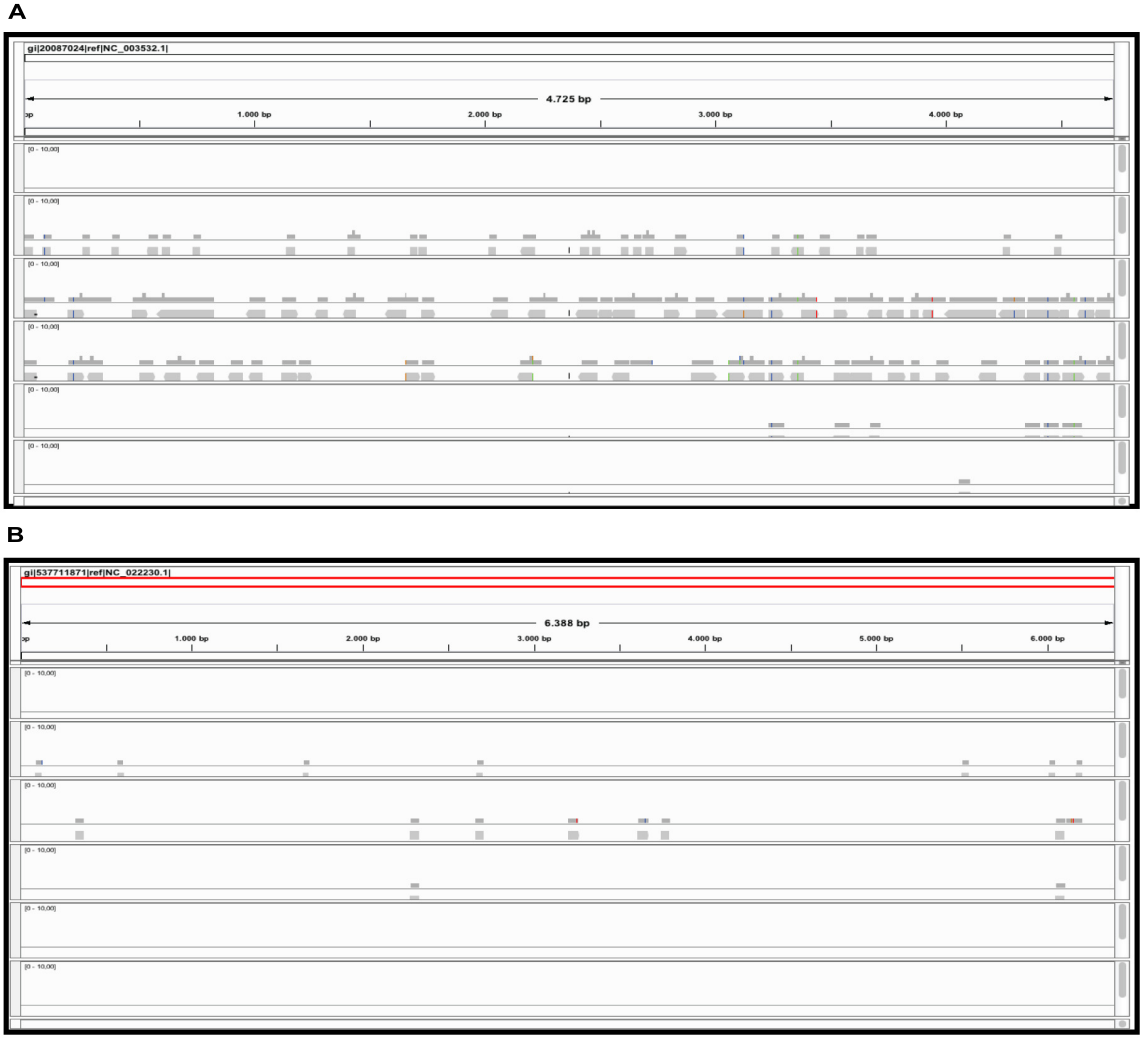
**Alignments of contig sequences obtained using Velvet REMRED, at different *k-*mer settings.** Contig distribution graph for CymRSV ref_seq **(A)** and ToMMV ref_seq **(B)** REMRED = Remove redundant.

The alignment of contigs to the *ToMMV* reference genome confirms the findings obtained for *CymRSV*; in **Figure 8A** we show that the *k* = 15 value remains the best setting to obtain highest number of contigs and also the highest coverage (i.e., 0,07, **Figure 8B**). Importantly, contigs obtained at a *k* value of 15 do not cover the exact same genome segments compared to those obtained with other *k*-mers (**Figure 9B**). This also holds for *CymRSV* analysis (**Figure 9A**). Thus, assemblies generated at different *k* values may complement each other to help the reconstruction of more complete viral genomes and increase the coverage as later discussed.

**FIGURE 10:**
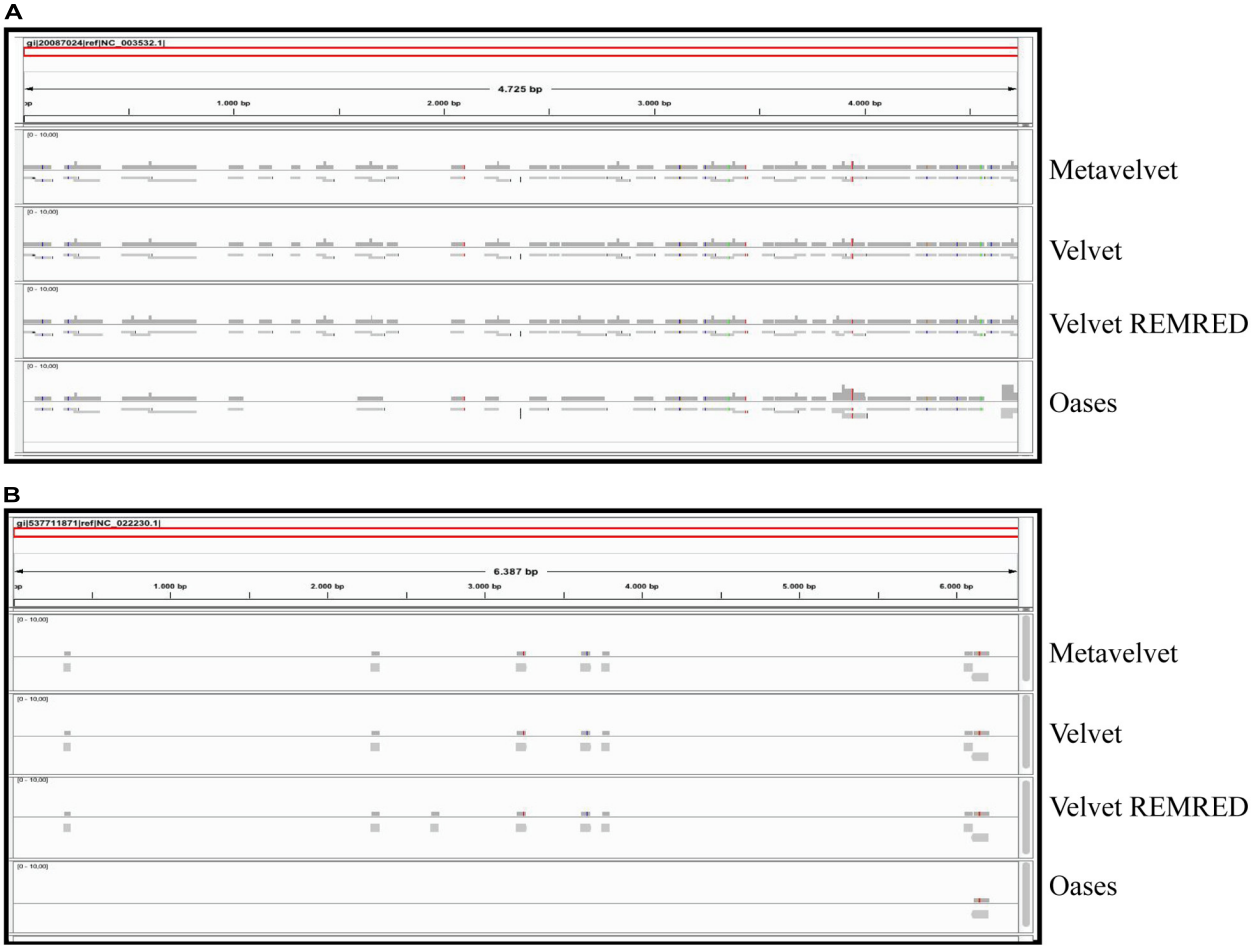
**Alignments of contig sequences obtained using Velvet, Metavelvet, and Oases short RNA assembly tools at *k* = 15.** Contig distribution graph for CymRSV ref_seq **(A)** and ToMMV ref_seq **(B)** REMRED = Remove redundant are displayed.

Obviously, a coverage value of 0,07 cannot be considered an acceptable coverage and shows the limitations of such approach for discovering novel viral entities. In **Figure 10** we compare contigs which are *de novo* assembled using different bioinformatics tools at a *k* = 15 settings in the cases of *CymRSV* and *ToMMV* (**Figures 10A,B** respectively). Here we graphically confirm the findings revealed in **Figure 7** and in Supplementary data 2 and 3. Indeed, the genome coverage of *ToMMV* is significantly lower compared to that of *CymRSV* (**Figures 7A,B**) and this is at least partly due to the abundance of viral-deriving siRNAs in the library (i.e., 364.590 vs. 1.909; **Tables 1** and **2**).

## DISCUSSION

Viral metagenomics surveys in plants have estimated that only small fractions of virus species are known. Stobbe and Roossinck (2014) have recently proposed the following classification of viruses found by metagenomics: (i) Known–known: virus species or isolates that are already known to be in the environment, (ii) Unknown–known: new virus species or isolates of a known family, or known viruses that have not been found previously in the surveyed environment, and (iii) Unknown–unknowns: viruses that are completely novel and share little to no sequence similarity with other known viruses. In this study we use 10 million sRNAs reads library (**Figure 1**) containing CymRSV v-siRNAs. The high coverage level of the virus is indicated by the fact that each nucleotide of the viral genomic ref_seq is represented on average 77 times in sRNAs. Subsequent variant calling showed the presence of a discrete number of SNPs (**Table 1**). Our data describe a typical example of metagenomics analysis of a “known–known” virus in a model-permissive system (i.e., *N. benthamiana*). In this case the ref_seq guided approach is able to reconstruct 99% of the genome whereas the *de novo* based approach is able to cover 69% of the genome. The gap between the two approaches is likely due to the heterogeneity within viral populations, the low and unbalanced genome coverage, and the rather short length of the siRNAs (around 21 bp on average). It is expected that the assembly would at least to some extent benefit from an increased sequencing depth (i.e., by generating 20 million sRNA reads instead of 10 million) although this would of course lead to additional costs for sequencing and data managing (Seguin et al., 2014). Also it should be mentioned that the genome coverage of the *de novo* assembly was calculated based on a reference alignment tool using strict parameters to allow only a few mutations. It is to be expected that more permissive alignment strategies such as BLAST (Altschul et al., 1990) can detect more homologous regions between the assembly and the reference, thus allowing the reconstruction of a more complete genome. However, there is a risk that the allowance of a higher number of mismatches will contemporarily lead to the inclusion of erroneously assembled regions. Further investigations and quality assessment is needed to address this issue.

In the case of short infectious entities such as HSVd, a more permissive alignment strategy obtained by introducing more mismatches in the alignment settings (i.e., 1, 2, or 3), may better be able to cover small gaps (**Figure 3**). However, the approach still leaves un-resolved gaps that could be associated with specific variants in the non-conserved domain of the viroid (Keese and Symons, 1985; Visvader and Symons, 1985). This interpretation fits with the fact that up-to-date no HSVd was reported in chickpea and therefore the entity here reported could be a novel HSVd variant.

The data obtained on *CymRSV* indicates that for “known–known” and some “Unknown–known” viruses the ref-seq approach may find practical (cost-effective) applications in particular for surveys of viruses for diagnosis in agro-ecosystems, plant population (e.g., old varieties) and single plant tissues/organs or for wider environmental studies of ecogenomics (Roossinck, 2011). Indeed, when applying the same pipeline to the sRNA library of a plant population constituting an old Chickpea variety we were able to reveal the presence of *ToMMV* (NC_022230), a putative novel *Tobamovirus* naturally infecting tomatoes in Mexico (Li et al., 2013). Note to worthy, we show through a metagenomics approach that the *ToMMV* is already present in Europe and that in can be hosted by *C. arietinum*. The ToMMV has been just proposed as a novel species of the *Tobamovirus* genus based on sequence similarity with other species of the genus. Phylogenetic analysis shows that *ToMMV* was clustered together with a group of Tobamoviruses mainly infecting solanaceous plants and therefore the presence in chickpea may give good reasons for further characterization of the viral genome, i.e., by generating a higher sRNA sequencing depth with the aim to increase the overall coverage. In cases where plant populations are studied classical molecular approaches are not always applicable. Indeed, an RT-PCR strategy was designed on assembled contigs and attempted on total RNA (see Materials and Methods) but no amplified products were observed. This may be due to the very low titer of virus in the tissues and/or to the infection of a discrete number of plants within the population composing the variety. The presence of a *Tobamovirus* into old varieties of Chickpea in Puglia is not surprising since this genus of plant viruses is known to be hosted by a wide range of plant species, including legumes. Moreover, all viral species are known to be transmitted mechanically and also through contaminations of the seed teguments (Broadbent, 1965).

In summary, a reference-guided approach appeared the most efficient in reconstructing viral metagenomes. Our results indicate that, using an appropriate short-read alignment mapping tool, even low abundant viruses can be well reconstructed. (e.g., at average sequencing depth lower than 7%, still 87% of the virus genome could be assembled). The *de novo* assembly based approaches reached a non-appreciable genome coverage and show a relatively high degree of fragmentation. Nonetheless the contigs generated were sufficiently long for assigning a proper taxonomic classification. Remarkably, the removal of duplicate sequences or the use of Metavelvet assembly software, which is specifically designed for metagenomics applications, did not contribute to more complete assemblies (i.e., longer contigs). At different *k*-mer settings all genome-based assembly strategies used yield similar genome coverage. In contrast, the use of a transcriptome-based method such as Oases resulted in longer contigs and may therefore be the method of choice for v-siRNA-based assemblies. Even if the total genome coverage was lower than in the case of genome-based assembly strategies, the increased average length may provide better anchor points for primer design of PCR products.

Thus, we deduce that transcriptome-based algorithms (i.e., those having adapted the original De Bruijn graph to assemble differentially expressed non-repetitive genes) could better manage the (large) differences between frequencies of sRNA reads covering the various viruses. Strategies based on the original De Bruijn graph algorithm instead attempt to remove *k*-mers with extreme abundance (also simulated by our REMRED approach). Moreover metagenomic-based assembly strategies need to overcome genome-repetitiveness in addition to differences in genome coverage: it may be that the complexity of these issues is higher than the v-siRNA assembly problem where genome repetitiveness is less of an issue.

We conclude that no method or particular *k*-mer setting was able to generate a full coverage, but also that different parameter settings led to assembly of unique (non-overlapping) v-siRNAs. Thus the use of a consensus-based strategy, where a master consensus genome is constructed from multiple assemblies (i.e., at different settings) could potentially be a more robust approach to reconstruct more complete viral genomes.

## Conflict of Interest Statement

The authors declare that the research was conducted in the absence of any commercial or financial relationships that could be construed as a potential conflict of interest.
