# Supplementary material for: Bioinformatics approaches for viral metagenomics in plants using short RNAs: model case of study and application to a Cicer arietinum population
Source: Front Microbiol. 2015 Jan 27;5:790. doi: 10.3389/fmicb.2014.00790 (PMC4307218; doi:10.3389/fmicb.2014.00790)
Supplement: Supplementary file 1 [file Data_Sheet_1.DOCX]

**Supplementary Data 1**. Outputs of contigs of Velvet, Metavelvet and Osases. REMRED= renove redundant.

A

| VELVET STANDARD | k11 | k13 | k15 | k17 | k19 | k21 |
| --- | --- | --- | --- | --- | --- | --- |
| Number of sequences | 16 | 10.260 | 16.604 | 6.998 | 1.832 | 304 |
| Sum (bp) | 348 | 283.764 | 780.039 | 421.193 | 125.910 | 24.619 |
| Max sequence size | 24 | 57 | 407 | 393 | 464 | 599 |
| Min sequence size | 21 | 25 | 29 | 33 | 37 | 41 |
| Average sequence size | 21 | 27 | 46 | 60 | 68 | 80 |

B

| VELVET_REMRED | k11 | k13 | k15 | k17 | k19 | k21 |
| --- | --- | --- | --- | --- | --- | --- |
| Number of sequences | 11 | 9.888 | 2.3251 | 7.693 | 2.099 | 302 |
| Sum (bp) | 240 | 274.234 | 1.000.599 | 452.113 | 139.738 | 2.3978 |
| Max sequence size | 24 | 57 | 228 | 393 | 464 | 601 |
| Min sequence size | 21 | 25 | 29 | 33 | 37 | 41 |
| Average sequence size | 21 | 27 | 43 | 58 | 66 | 79 |

C

| METAVELVET | k11 | k13 | k15 | k17 | k19 | k21 |
| --- | --- | --- | --- | --- | --- | --- |
| Number of sequences | 16 | 1.0260 | 1.6604 | 5.270 | 2.453 | 344 |
| Sum (bp) | 348 | 283.764 | 780.039 | 328.478 | 158.555 | 27.020 |
| Max sequence size | 24 | 57 | 407 | 393 | 374 | 599 |
| Min sequence size | 21 | 25 | 29 | 33 | 37 | 41 |
| Average sequence size | 21 | 27 | 46 | 62 | 64 | 78 |

D

| OASES | k11 | k13 | k15 | k17 | k19 | k21 |
| --- | --- | --- | --- | --- | --- | --- |
| Number of sequences | 0 | 39 | 2.337 | 1.907 | 662 | 171 |
| Sum (bp) | 0 | 4.405 | 234.271 | 190.089 | 69.268 | 19.972 |
| Max sequence size | 0 | 147 | 530 | 919 | 740 | 604 |
| Min sequence size | 0 | 26 | 65 | 67 | 69 | 71 |
| Average sequence size | 0 | 112 | 100 | 99 | 104 | 116 |

**Supplementary Data 2**: Alignment of contigs to CymRSV ref_seq. REMRED= renove redundant.

A

Velvet

|  | k11 | k13 | k15 | k17 | k19 | k21 |
| --- | --- | --- | --- | --- | --- | --- |
| # Position in reference (NC_003532) | 4733 | | | | | |
| # Aligned contigs vs CymRSV (NC_003532) | 0 | 38 | 6 | 1 | 27 | 41 |
| # Nucleotides in consensus sequence | 0 | 924 | 3.247 | 2.266 | 385 | 47 |
| Fraction of reference covered | 0 | 0,2 | 0,69 | 0,48 | 0,08 | 0,01 |
| Number of SNPs with NC_003532 | 0 | 2 | 14 | 12 | 2 | 0 |

B

Velvet RemRed

|  | k11 | k13 | k15 | k17 | k19 | k21 |
| --- | --- | --- | --- | --- | --- | --- |
| # Position in reference (NC_003532) | 4733 | | | | | |
| # Aligned contigs vs CymRSV (NC_003532) | 0 | 30 | 45 | 39 | 6 | 1 |
| # Nucleotides in consensus sequence | 0 | 1.031 | 3.246 | 2.311 | 385 | 47 |
| Fraction of reference covered | 0 | 0,22 | 0,69 | 0,49 | 0,08 | 0,01 |
| Number of SNPs with NC_003532 | 0 | 2 | 14 | 11 | 2 | 0 |

C

Metavelvet

|  | k11 | k13 | k15 | k17 | k19 | k21 |
| --- | --- | --- | --- | --- | --- | --- |
| # Position in reference (NC_003532) | 4733 | | | | | |
| # Aligned contigs vs CymRSV (NC_003532) | 0 | 27 | 41 | 34 | 6 | 2 |
| # Nucleotides in consensus sequence | 0 | 924 | 3.247 | 2.115 | 385 | 125 |
| Fraction of reference covered | 0 | 0,2 | 0,69 | 0,45 | 0,08 | 0,03 |
| Number of SNPs with NC_003532 | 0 | 2 | 14 | 10 | 2 | 1 |

D

Oases

|  | k11 | k13 | k15 | k17 | k19 | k21 |
| --- | --- | --- | --- | --- | --- | --- |
| # Position in reference (NC_003532) | 4733 | | | | | |
| # Aligned contigs vs CymRSV (NC_003532) | 0 | 1 | 13 | 17 | 2 | 1 |
| # Nucleotides in consensus sequence | 0 | 73 | 2.692 | 1.182 | 150 | 78 |
| Fraction of reference covered | 0 | 0,02 | 0,57 | 0,25 | 0,02 | 0,02 |
| Number of SNPs with NC_003532 (Nucmer) | 0 | 1 | 12 | 3 | 1 | 1 |

**Supplementary Data 3**: Alignment of contigs to ToMMV ref_seq. REMRED= renove redundant.

A

Velvet

|  | k11 | k13 | k15 | k17 | k19 | k21 |
| --- | --- | --- | --- | --- | --- | --- |
| # Position in reference (NC_022230) | 6398 | | | | | |
| # Aligned contigs vs TMMV (NC_022230) | 0 | 7 | 7 | 1 | 0 | 0 |
| # Nucleotides in consensus sequence | 0 | 245 | 418 | 53 | 0 | 0 |
| Fraction of reference covered | 0 | 0,04 | 0,07 | 0,01 | 0 | 0 |
| Number of SNPs with NC_022230 | 0 | 0 | 2 | 0 | 0 | 0 |

B

Velvet RemRed

|  | k11 | k13 | k15 | k17 | k19 | k21 |
| --- | --- | --- | --- | --- | --- | --- |
| # Position in reference (NC_022230) | 6398 | | | | | |
| # Aligned contigs vs TMMV (NC_022230) | 0 | 7 | 8 | 2 | 0 | 0 |
| # Nucleotides in consensus sequence | 0 | 248 | 467 | 107 | 0 | 0 |
| Fraction of reference covered | 0 | 0,04 | 0,07 | 0,02 | 0 | 0 |
| Number of SNPs with NC_022230 | 0 | 0 | 2 | 0 | 0 | 0 |

C

Metavelvet

|  | k11 | k13 | k15 | k17 | k19 | k21 |
| --- | --- | --- | --- | --- | --- | --- |
| # Position in reference (NC_022230) | 6398 | | | | | |
| # Aligned contigs vs TMMV (NC_022230) | 0 | 7 | 7 | 0 | 0 | 0 |
| # Nucleotides in consensus sequence | 0 | 245 | 418 | 0 | 0 | 0 |
| Fraction of reference covered | 0 | 0,04 | 0,07 | 0 | 0 | 0 |
| Number of SNPs with NC_022230 | 0 | 0 | 2 | 0 | 0 | 0 |

D

Oases

|  | k11 | k13 | k15 | k17 | k19 | k21 |
| --- | --- | --- | --- | --- | --- | --- |
| # Position in reference (NC_022230) | 6398 | | | | | |
| # Aligned contigs vs TMMV (NC_022230) | 0 | 0 | 1 | 0 | 0 | 0 |
| # Nucleotides in consensus sequence | 0 | 0 | 98 | 0 | 0 | 0 |
| Fraction of reference covered | 0 | 0 | 0,02 | 0 | 0 | 0 |
| Number of SNPs with NC_022230 | 0 | 0 | 2 | 0 | 0 | 0 |
